# Comprehensive Assessment of Incidence, Risk Factors, and Mechanisms of Impaired Medical and Psychosocial Health Outcomes among Adolescents and Young Adults with Cancer: Protocol of the Prospective Observational COMPRAYA Cohort Study

**DOI:** 10.3390/cancers13102348

**Published:** 2021-05-13

**Authors:** Olga Husson, Marjolijn J. L. Ligtenberg, Lonneke V. van de Poll-Franse, Judith B. Prins, Martin J. van den Bent, Mies C. van Eenbergen, Renske Fles, Eveliene Manten-Horst, Jourik A. Gietema, Winette T. A. van der Graaf

**Affiliations:** 1 Department of Medical Oncology, Netherlands Cancer Institute, 1066 CX Amsterdam, The Netherlands; R.Fles@nki.nl (R.F.); w.vd.graaf@nki.nl (W.T.A.v.d.G.); 2 Division of Psychosocial Research and Epidemiology, Netherlands Cancer Institute, 1066 CX Amsterdam, The Netherlands; l.vd.poll@nki.nl; 3 Division of Clinical Studies, Institute of Cancer Research, London SM2 5NG, UK; 4 Department of Human Genetics, Radboud University Medical Center, 6525 GA Nijmegen, The Netherlands; Marjolijn.Ligtenberg@radboudumc.nl; 5 Department of Pathology, Radboud University Medical Center, 6525 GA Nijmegen, The Netherlands; 6 Research & Development, Netherlands Comprehensive Cancer Organization (IKNL), 3511 DT Utrecht, The Netherlands; M.vanEenbergen@iknl.nl; 7 Department of Medical and Clinical Psychology, Tilburg University, 5037 AB Tilburg, The Netherlands; 8 Department of Medical Psychology, Radboud Institute for Health Sciences, Radboud University Medical Centre, 6525 GA Nijmegen, The Netherlands; Judith.Prins@radboudumc.nl; 9 Department of Neurology, Erasmus University Medical Center, 3015 GD Rotterdam, The Netherlands; m.vandenbent@erasmusmc.nl; 10 Dutch AYA Care Network, 3511 DT Utrecht, The Netherlands; e.manten-ayanationaal@iknl.nl; 11 Department of Medical Oncology, University Medical Center Groningen, 9713 GZ Groningen, The Netherlands; j.a.gietema@umcg.nl; 12 Department of Medical Oncology, Erasmus MC Cancer Institute, Erasmus University Medical Center, 3015 GD Rotterdam, The Netherlands

**Keywords:** adolescent and young adult oncology, late effects, health-related quality of life, survival, genetic risk

## Abstract

**Simple Summary:**

Adolescents and young adults (AYA), aged 18–39 years at first cancer diagnosis, are recognized as a distinct population within the oncology community due to the unique challenges they encounter including recognition, diagnosis, treatment, and monitoring of their disease. It is imperative for advances in the field of AYA oncology to pool data sources (patient-reported outcomes, clinical, treatment, genetic, and biological data) across institutions and countries and create large cohorts that include the full range of AYA ages and diagnoses to be able to address the many pressing questions that remain unanswered in this vulnerable population. The Dutch COMPRAYA study aims to examine the incidence, risk factors, and mechanisms of impaired health outcomes (short- and long-term medical and psychosocial effects) over time among AYA cancer patients. The overarching aim is to provide a research infrastructure for (future) data analyses and observational retrospective/prospective ancillary studies and to expand data collection to other countries.

**Abstract:**

Adolescent and young adult (AYA) cancer patients suffer from delay in diagnosis, and lack of centralized cancer care, age-adjusted expertise, and follow-up care. This group presents with a unique spectrum of cancers, distinct tumor biology, cancer risk factors, developmental challenges, and treatment regimens that differ from children and older adults. It is imperative for advances in the field of AYA oncology to pool data sources across institutions and create large cohorts to address the many pressing questions that remain unanswered in this vulnerable population. We will create a nationwide infrastructure (COMPRAYA) for research into the incidence, predictive/prognostic markers, and underlying mechanisms of medical and psychosocial outcomes for AYA between 18–39 years diagnosed with cancer. A prospective, observational cohort of (*n* = 4000), will be established. Patients will be asked to (1) complete patient-reported outcome measures; (2) donate a blood, hair, and stool samples (to obtain biochemical, hormonal, and inflammation parameters, and germline DNA); (3) give consent for use of routinely archived tumor tissue and clinical data extraction from medical records and registries; (4) have a clinic visit to assess vital parameters. Systematic and comprehensive collection of patient and tumor characteristics of AYA will support the development of evidence-based AYA care programs and guidelines.

## 1. Introduction

Adolescents and young adults (AYA) are recognized as a distinct population within the oncology community due to the unique challenges they encounter including recognition, diagnosis, treatment, and monitoring of their disease [1,2,3]. The US National Cancer Institute proposed defining AYA as those aged 15–39 years at diagnosis [2], however it also concluded that this age range should be flexibly applied, depending on the health care delivery system [4]. In the Netherlands, there is a clear distinction between centralized pediatric oncology (0–18 years), and adult oncology (18 years of age and older). Patients diagnosed with cancer between 18 and 39 years old were often called the “lost tribe” as they are too old to profit from the integrated pediatric care [5,6] and, consequently, are treated in the adult health care system in many different hospitals throughout the country.

Although cancer is a disease primarily affecting older adults, around 3800 AYAs are diagnosed with cancer in the Netherlands every year, which is around 5% of all invasive cancer diagnoses [7]. Over the previous decades, the incidence of cancer among AYAs has slightly increased, for some tumors relatively more common than among older adults [8]. AYAs typically present with a rare tumor or a common tumor at an unusual age: either with a pediatric malignancy (e.g., acute lymphoblastic leukemia, pediatric brain tumors), a tumor of AYA age (e.g., Hodgkin’s disease, melanoma, germ cell, or thyroid cancer) or, with an adult tumor (e.g., gastrointestinal, lung, or breast carcinomas) [9]. Over 80% of AYAs with cancer will survive long-term (five or more years) [3,10]. Nevertheless, population-based cancer registry data have generally shown poorer survival of AYAs compared with children and older adults [11,12]. Nonetheless, more recently, decreases in mortality rates for all AYAs combined were found, although with notable differences by tumor type [11,13,14,15,16].

Advances in cancer treatment have led to increased survival rates, however, even if patients survive and are cured, they are still at risk of the development of long-term medical (e.g., cardiovascular disease or second malignancies) or psychosocial (e.g., high distress levels [17], financial toxicity due to unemployment without a prior career job) long-term and late effects of cancer and its treatment, that may also increase the risk of late mortality [18,19,20,21,22]. The impact and consequences cancer has on AYA, including the gap in survival outcomes, differs from other age groups for several reasons [23]:

- Insufficient awareness of cancer risk and symptoms among AYA and healthcare professionals resulting in prolonged diagnostic trajectories [24,25];
- Unique and incompletely understood tumor biology—cancers that are histologically indistinguishable across the age spectrum may be characterized by particular biological features in the AYA population [9,26,27];
- Distinct age-related physiology, pharmacology, and genomic properties with respect to cancer susceptibility and treatment [9];
- Unequal access to and low participation rates in clinical trials [1];
- Lack of age-adjusted treatments [28] and age-specific psychosocial care—”I am treated like my 74-year-old grandma” [29,30];
- Adolescence and emerging and young adulthood are complex phases of life due to the many physical, emotional, cognitive, and social transitions [31]. Important developmental tasks need to be achieved, such as forming one’s own identity and a healthy body image, establishing autonomy, responsibility and independence, finishing education and starting a career, starting a relationship and having children [31]. A cancer diagnosis challenges AYAs’ abilities to achieve these developmental milestones [32,33]. The way in which AYA cancer patients adjust to their cancer experience might have life-long implications for the quality of their survival [34,35,36,37].

Fortunately, the identification of the gaps in AYA survival outcomes with mainly population-based registry data has prompted efforts to expand the availability of clinical trials and to lower barriers for trial participation for AYA. It also enforced the development of more effective treatment regimens and evidence-based secondary prevention surveillance strategies, and the start of a broad range of needed AYA care programs globally [29].

In the Netherlands, a nationwide AYA ‘Young and Cancer’ Care Network was introduced in 2014 to enable stepwise, structured, age-adjusted AYA care across hospitals. The majority of AYA cancer patients are treated at one of the eight university medical centers or the Netherlands Cancer Institute. AYA nurse-led specialist care and AYA outpatient services have been introduced next to tumor-specific medical services (https://ayazorgnetwerk.nl/, accessed on 12 April 2021).

Nevertheless, the available population-based registries still do not incorporate complete clinical or biological information, nor do they necessarily include nonlethal treatment failures, incident comorbidities, and treatment-related sequelae, or patient-reported outcomes. In childhood cancer, clinical cohort studies overcome many of the limitations of registries. For example, within the Childhood Cancer Survivor Study (CCSS) detailed data of 35,923 childhood cancer survivors diagnosed between 1970 and 1999 were collected on cancer diagnosis, therapy received, and health- and quality-of-life-related outcomes [38]. Unfortunately, the CCCS cohort does not include patients aged 21 years and older at time of diagnosis, limiting its utility for AYA oncology. The DCOG-LATER cohort includes over 6000 five-year childhood cancer survivors diagnosed during 1963–2001 before age 18 years in the Netherlands [39]. Given the age-related differences in tumor epidemiology and biology, the developmental challenges and access to care, findings derived from childhood or older cancer cohorts cannot be extrapolated to AYAs. (Inter)nationally, large cohort studies exist that address many relevant long-term cancer-related health issues, but all from the perspective of tumor histology, rather than an AYA age-specific perspective.

It is imperative for advances in the field of AYA oncology to pool data sources (patient-reported outcomes, clinical, treatment, genetic, and biological data) across institutions and countries and create large cohorts that include the full range of AYA ages and diagnoses to be able to address the many pressing questions that remain unanswered in this vulnerable population. Globally, so far, the identification of AYA cancer patient subgroups that might be more susceptible to poor (age-specific) health outcomes has not been addressed in a systematic and coordinated way. The role of sociodemographic and treatment-associated risks, external exposures (e.g., lifestyle) and host factors (e.g., genetic, biological, physiological); or combinations of influences for impaired (age-specific) health outcomes, remains largely unknown. Understanding *who* is at risk and *why* will support the development of evidence-based AYA prevention, treatment and supportive care programs and guidelines.

The COMPRAYA study aims to examine the incidence, risk factors, and mechanisms of impaired health outcomes (short- and long-term medical and psychosocial effects and late effects) over time among AYA cancer patients in the Netherlands. The overarching aim of the COMPRAYA study is to provide a research infrastructure for (future) data analyses and observational retrospective and prospective ancillary studies and to expand data collection to other countries.

## 2. Methods and Analysis

The COMPRAYA study is a Dutch, nationwide, prospective, population-based, observational cohort study among AYA cancer patients.

### 2.1. Objectives

#### 2.1.1. Primary Objective

- To identify individual, environmental, biological, and psychological characteristics of AYA cancer patients who are at high risk for impaired medical and psychosocial health outcomes. In other words, to develop a prediction model for impaired medical and psychosocial health outcomes (at baseline and at 2-, 5- and 10-year follow-up).

#### 2.1.2. Secondary Objective(s)

- To assess the incidence of impaired (age-specific) medical (e.g., second tumor) and psychosocial (e.g., social isolation) health outcomes at each time-point (at baseline and at 2-, 5- and 10-year follow-up). We aim to compare some of the patient-reported outcomes and medical registry outcomes of AYA with those of control groups available to get a better idea of the impact of cancer on AYA.

#### 2.1.3. Exploratory Objective(s)

- To analyze the course of medical and psychosocial health outcomes over time (all timepoints needed);
- To analyze mediating mechanisms associated with impaired health outcomes in AYA cancer patients (at baseline and at 2-, 5- and 10-year follow-up).

#### 2.1.4. Other Objective(s)

- To form a prospective observational cohort of patients diagnosed with cancer at AYA age, and follow them over time until death.

### 2.2. Study Population

All AYAs diagnosed (18–39 years at primary diagnosis) with cancer (any type) within the first three months after diagnosis in one of the participating centers (all eight university medical centers and one cancer-specific hospital) in the Netherlands are eligible to participate in the COMPRAYA study. The presence of AYA services in these hospitals will enable the recruitment of patients across the participating centers.

#### 2.2.1. Inclusion Criteria

In order to be eligible to participate in this study, a subject must meet all of the following criteria:

- Pathological-confirmed cancer diagnosis;
- Age 18–39 years at time of first cancer diagnosis;
- Able to understand the informed consent form;
- Willing to provide written informed consent.

#### 2.2.2. Exclusion Criteria

- Mentally incompetent patients based on the opinion of treating physician;
- Inability to understand the Dutch language;
- Life expectancy less than 6 months based on the opinion of treating physician. Patients living with an uncertain or poor cancer prognosis have the option to participate in the INVAYA study. This qualitative interview study primarily aims to get a better understanding of the experiences and needs of AYA with life-limiting cancer in daily life and in the healthcare system, through the lens of the AYA themselves, the informal caregiver, and the healthcare professional [16].

### 2.3. Data Collection

Every 2 weeks, the local COMPRAYA research nurse will receive a list, derived from the Netherlands Cancer Registry (NCR; which routinely collects data of all individuals newly diagnosed with cancer), of AYAs diagnosed with cancer approximately 0–3 months ago. Eligible AYAs are preferably included before initiation of cytotoxic treatment.

The local COMPRAYA research nurse will receive the patient’s hospital number, if known, otherwise name and date of birth derived from the NCR. The local COMPRAYA research nurse, in consultation with the treating physicians, will check the patient selection on eligibility criteria and invite eligible patients for the study, on behalf of their treating physician. In case the local team identifies eligible patients before the list from the NCR has been received they are also allowed to invite patients to participate in the study. AYA cancer patients will receive an invitation package by mail consisting of a letter, patient information and informed consent. The local COMPRAYA research nurse will contact the patients one week after sending the invitation package to give more information if necessary, check whether they want to participate, and schedule an appointment at the outpatient clinic (preferably simultaneously with other routine appointments). A unique personal study number will be assigned to each AYA participant.

Participants will receive an information letter detailing how to fill in questionnaires via PROFILES (patient-reported outcomes following initial treatment and long-term evaluation of survivorship) [40]. PROFILES is a web-based system for collecting patient-reported outcomes in cancer research. The invitation letter includes a link to a secure website (www.profielstudie.nl, accessed on 12 April 2021), a login name, and a password. If the patient prefers written rather than digital communication, paper questionnaires can be completed and returned to the researcher using a return envelope. 

Once the AYA cancer patient has consented to participate and has completed all the planned assessments at baseline, we will follow them prospectively by annually sending a selection of questionnaires. Collection of clinical and treatment data and samples (e.g., feces, hair, and blood) will be done at baseline and at two, and five years after diagnosis (Table 1). Patients can voluntarily withdraw from the study at any moment in time. If a patient loses capacity to consent during the study, their existing data will be stored for use in the study but no further measurements will be performed. The study will be conducted according to the guidelines of the Declaration of Helsinki and will be in line with the Dutch/European privacy legislation (AVG/GDPR). The study protocol was approved by the Medical Research Ethics Committee Netherlands Cancer Institute—The Antoni van Leeuwenhoek Hospital on 9 November 2020 (NL73969.031.20). Recruitment has been delayed due to the COVID-19 pandemic and is anticipated to start between March and May 2021. The data management plan of COMPRAYA is available upon request.

### 2.4. Measures

**Figure 1:**
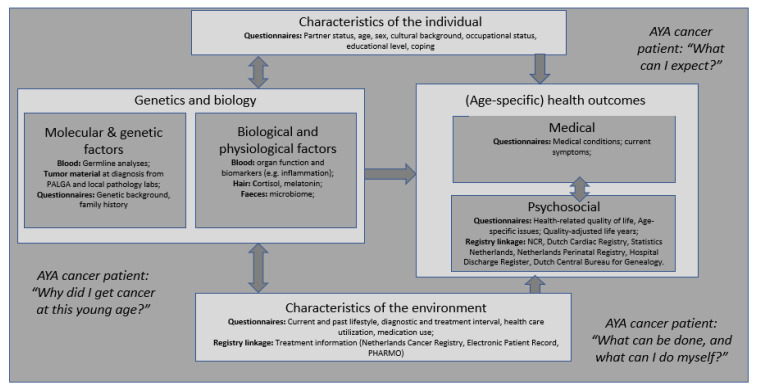
Conceptual model of COMPRAYA study. Revised Wilson and Cleary Model [41,42,43].

The revised Wilson and Cleary conceptual model of patient outcomes (Figure 1) [41,42,43] was used for the selection of appropriate measurement parameters. The authors of the model present it as taxonomy of patient health outcomes that are linked with *characteristics of the individual, characteristics of the environment, and biological and physiological* variables.

#### 2.4.1. Medical and Psychosocial Health Outcomes (Questionnaires and Registries)

##### AYA Impact of Cancer

To identify the positive and negative psychosocial impact of cancer, participants will complete a modified version of the 18-item Life Impact Checklist [44,45] at each time-point. Nine of the 18 items are from the original checklist [45], and nine items related to other life domains that have been identified as important to AYA cancer patients (body image, future goal setting, and plans for education and work) will be included [46,47].

##### Health-Related Quality of Life

The EORTC QLQ-C30 is a 30-item HRQoL questionnaire consisting of five functional scales (physical, role, cognitive, emotional, and social), a global quality-of-life scale, three symptom scales (fatigue, pain, nausea, and vomiting) and a number of single items assessing common symptoms (dyspnea, loss of appetite, sleep disturbance, constipation, and diarrhea) and the perceived financial impact of the disease [48]. 

The EQ-5D-5L is a descriptive system for the measurement of health. It measures HRQoL on five dimensions of health—mobility, self-care, usual activities, pain-discomfort, and anxiety/depression [49].

Both questionnaires will be part of the questionnaire package at each time-point.

##### Psychological Distress

Psychological distress will be assessed at each time-point with the Hospital Anxiety and Depression Scale (HADS), with seven items each for assessing symptoms of anxiety and depression [50].

##### Medical History/Conditions

Medical history/conditions will be assessed with a short version of the questionnaire used in the St. Jude Childhood Cancer Survivorship study (e.g., hearing/vision/speech, urinary, hormonal, heart and circulatory, respiratory, digestive, brain and nervous systems, child births and malignancies). At baseline medical history is assessed by the local COMPRAYA research nurse; at 2-, 5- and 10-year follow-up all changing medical conditions will be registered via the patient-reported questionnaire.

##### Costs Related to Productivity and Medical Consumption

The Productivity Cost Questionnaire (iPCQ) will be used to evaluate the impact of disease on the productivity of a person and the Medical Consumption Questionnaire (iMCQ) will gain more insight in the frequently occurring contacts with health care providers [51]. The combination of these two questionnaires will be used for the measurement of costs.

The Productivity and Disease Questionnaire (PRODISQ) will be used to assess all relevant aspects of the relationship between health and productivity including absence from work, compensation mechanisms that may reduce productivity loss, reduced productivity at work (efficiency losses), and productivity costs at the level of organizations [52].

Answers on the questionnaires will be registered by the local COMPRAYA research nurse on baseline and the questionnaires will be completed by patients themselves at each follow-up assessment.

##### Registry Linkage

Linkage will be done with the NCR (second malignancies), Personal Records Database (survival status), Dutch Cardiac Registry (cardiac interventions, hospitalizations), Statistics Netherlands (cause-of-death, work-related outcomes including individual income, type of occupation, number of working hours, number of contracts, receipt of disability benefits, unemployment benefits, and welfare), the Netherlands Perinatal Registry (pregnancy outcomes and children) and the Hospital Discharge Register (hospitalizations and referrals).

#### 2.4.2. Characteristics of the Individual and Environment

Sociodemographic data including age, gender, marital status, children, education, and breadwinner status (sole/shared) will be obtained via the local COMPRAYA research nurse at baseline and all changes at follow-up will be registered via the patient-reported questionnaire. 

##### Clinical and Health Care Characteristics (Registries, Medical Records, and Questionnaires)

Clinical information including cancer site, size, stage, grade, pathological stage, month/year of diagnosis, received (and future) treatment(s), i.e., surgery, radiation, chemotherapy, immunotherapy, targeted treatment, hormonal therapy or combination, medication, recurrence(s) of the disease, adverse events (adapted CTC), comorbidity, and fertility preservation will be obtained from the medical records, the NCR, and/or via self-report. The data will be registered in an eCRF.

Data on drug use will be obtained from the PHARMO database (which is already linked to the NCR). The drug-dispensing database of PHARMO contains complete longitudinal information on the dispensed drug, prescriber, date and amount of dispensing, prescribed dose regimens, and thus, on duration of drug use obtained from community pharmacies [53]. 

Questions on diagnostic and treatment interval [54,55] will be registered by the local COMPRAYA research nurse at baseline. Questions on clinical trial participation, use of over-the-counter medication and complementary and alternative medicine will be registered by the local COMPRAYA research nurse at baseline and added to the questionnaire package at all follow-up assessments.

#### 2.4.3. Psychosocial Characteristics (Questionnaire)

##### Coping Style

At baseline and at 2-, 5- and 10-year follow-up, coping will be assessed with the Cognitive Emotion Regulation Questionnaire (CERQ) [56]. The CERQ is a multidimensional questionnaire constructed in order to identify the cognitive coping strategies someone uses after having experienced negative events or situations. Contrary to other coping questionnaires that do not explicitly differentiate between an individual’s thoughts and his or her actual actions, the present questionnaire refers exclusively to an individual’s thoughts after having experienced a negative event.

##### Resilience

At baseline and at 2-, 5- and 10-year follow-up, resilience will be assessed by the Brief Resilience Scale (BRS) [57]. Resilience is a skill which helps people to recover from a life event. People with high (perceived) resilience have the ability to move on faster after a setback.

##### Illness Perceptions

At baseline and at 2-, 5- and 10-year follow-up, illness perceptions will be assessed using the Brief Illness Perception Questionnaire (B-IPQ), a nine-item instrument used to assess cognitive and emotional representations of the illness [58].

##### Autonomy

At each time-point, the short (four-item) version of the Patient Autonomy Questionnaire (PAQ) will be added to the questionnaire package to assess autonomy problems [59].

##### Spirituality

At baseline and at 2-, 5- and 10-year follow-up, the 25-item SPIRIT questionnaire will be used to measure spirituality in five dimensions—experiential, cognitive, coping, moral, and social [60]. 

#### 2.4.4. Lifestyle and Other Environmental Exposures (Questionnaire)

Questions from the American National Institute of Health studies will measure lifetime smoking history and current smoking status, lifetime alcohol consumption history and current alcohol consumption, history of and current recreational drug/substance use, history of previous illnesses, disorders, lifetime physical activity history, and past and present sunbathing behavior. The Short Questionnaire to Assess Health-Enhancing Physical Activity (SQUASH) will be used to assess physical activity [61]. The local COMPRAYA research nurse will register the answers of the patients at baseline, patients will complete questionnaires on current lifestyle behavior at each time-point by themselves.

Food intake will be assessed by asking patients to register all foods and drinks they had taken during the day using the online ‘Eetmeter’ from the Dutch ‘Voedingscentrum’. Participants will be asked to complete the Eetmeter at baseline and at 1-, 2-, 5-, and 10-year follow-up for three consecutive days (including one day in the weekend). The Eetmeter is connected to the Dutch Nutrients Database (NEVO) so the quantity of macro- and micronutrients will be calculated immediately. Patients participating in the study will receive a link to create an account for the Eetmeter. Patients will get access to their results if they indicate they would like to receive these.

#### 2.4.5. Genetics and Biology

##### Genetic Background and Family History (Questionnaire)

For synoptic reporting of the occurrence of cancer in first- and second-degree relatives, the standard list of university medical centers (outpatient hereditary cancer clinics) will be used.

##### Special Phenotypic Features of the Patient (Questionnaire)

As genetic cancer predisposition may also be associated with other phenotypic features a questionnaire is made to synoptically report features, e.g., head circumference, skin lesions, cleft lip, an aberrant number of fingers or toes, number of miscarriages. 

##### Tumor Material (PALGA Linkage)

Patients are asked to provide consent for use of tumor and normal material, and data that is already registered in the nationwide PALGA system. In addition, participants are asked to provide consent for the use of tissue material of recurrences or new malignancies, and data concerning these new events.

##### Blood (Hospital Visit)

At baseline and at 2 and 5 years, blood of AYA cancer patients will be collected during a (regular follow-up) visit after an overnight fasting by venipuncture at one of the participating hospitals using a standard protocol (accompanied with a standard questionnaire). 

To minimize possible differences in the processing of the samples for analysis, the blood will be processed according to the COMPRAYA standard operating procedures and temporarily stored in the participating hospitals. On a regular basis, the blood will be collected from the hospitals and analyzed and stored in line with our research questions at the Netherlands Cancer Institute (sponsor).

Study blood collection serves two purposes:

(1) Germline DNA for research on cancer susceptibility genes, SNP-array, methylation profile, and telomere length (separate informed consent from patient for this part);
(2) Biomarkers of impaired health outcomes (metabolic syndrome, markers of inflammation, fertility hormones, methylation profile, markers of the senescence-associated secretory phenotype (SASP), biochemical markers for cardiovascular damage, and telomere length assessed in white blood cell DNA as a measure of ageing).

##### Feces

At baseline and at 2 and 5 years, AYA cancer patients from three participating sites will be asked to collect feces samples at home the day before, or the day of assessment/blood collection (accompanied with a standard questionnaire). These samples will be used to evaluate exploratory biomarkers (microbiome), which may be predictive for impaired medical health outcome endpoints.

##### Hair

Cortisol measurement in hair is a relatively new method that allows for assessments of chronic stress over time. Levels of melatonin can be detected in blood and saliva, but, similar to cortisol, melatonin is also stored in the hair, allowing for stable assessments of melatonin production over extended periods of time. Melatonin can act as an indicator for prolonged sleep disruption [62]. 

At baseline and at 2 and 5 years, hair sampling will be done by the local COMPRAYA research nurse in the hospital. 

#### 2.4.6. Physiological Characteristics (Hospital)

Physical examination during a hospital visit will include: standardized resting blood pressure and heart rate, body composition (BMI (height, weight, waist-, hip-, and calf circumference)), bioimpedance, and grip strength.

##### Bioimpedence

Whole-body single frequency (SF) (50 kHz) BIA measurements (impedance, resistance, reactance, and phase angle) will be performed according to standard operating procedures by the local COMPRAYA research nurse, using Bodystat 500 (Bodystat Ltd., Isle of Man, British Islands).

##### Grip Strength (GS)

All handgrip tests will be executed with the dominant hand using the Jamar system (Lafayette Instrument, Lafayette, IN, USA), consisting in an adjustable handgrip handle (standard JD configuration) equipped with in-built compression load cell and connected via a strain-gauge amplifier to a computer. Patients will be asked to squeeze the handle as hard as possible three times with 30 s interval. Afterwards, subjects will be instructed to maintain this maximal pressure as long as possible. The time (in seconds) during which GS dropped to 50% of its maximum will be recorded as FR (time to fatigue) and the maximal grip strength value reached during the test as GS max.

### 2.5. Statistical Analysis and Power Calculation

#### 2.5.1. Sample Size

Recruitment will take place over a 4.5-year period. With an incidence of 3800 new AYA cancer diagnoses/year, 1-year survival of 93%, nationwide coverage of 60% by participating hospitals, and response rate of 40%, we aim to recruit 4000 AYA cancer patients in this period. 

The sample size needed for regression analyses for our primary aim is based on the rule of thumb of Green (book-discovering statistics using SAS, Andy Field, 2010, page 197 [63]). There are two rules, one for the minimum number for optimal fit of the model and one to test the individual predictors. The minimum number for optimal fit is 50 + 8 k (k = number of predictors). If we assume 25 predictors (characteristics of the individual (e.g., age, sex, cultural background, partner status, educational level, tumor type, disease stage, comorbid conditions), characteristics of the environment (e.g., cancer treatment, lifestyle, and genetic and biological factors) in the model, then we need 50 + 200 = 250 patients. The minimum number for individual predictors is 104 + k = 129.

The secondary endpoint will be describing the proportion of patients with an impaired score for each health outcome at each time-point.

No formal sample size calculation is being conducted for the exploratory objectives. This project is designed to continuously include new AYA cancer patients (depending on funding) and will provide an on-going source of patient data to answer secondary research questions.

#### 2.5.2. Statistical Analyses

All statistical analyses will be performed using R software (https://www.r-project.org/, accessed on 12 April 2021). All statistical tests will be two-sided and differences are considered significant if *p* < 0.05. Missing items in the questionnaire will be handled as follows: If (an) item(s) from a multi-item scale is/are missing, and at least half of the items from the scale have been answered, then scale scores are calculated ignoring any items with missing values, which is the same as assuming that the missing items have values equal to the average of those items. If fewer than half of the items from the scale have been answered, then the scale score is set to missing. For single-item measures, score is also set to missing.

##### Primary Endpoint

Univariable and multivariable linear (continuous outcome) and logistic (dichotomous outcome) regression analyses will be performed to test the association between potential risk factors (characteristics of the individual, environment, and genetic and biological factors) and age-specific outcomes (as mentioned above). The multivariable model will consist of variables statistically significant in univariable analysis. A backward selection method will be used to identify a parsimonious prediction model per outcome. Bonferroni correction for multiple testing will be applied.

##### Secondary

Descriptive statistics (mean, median, standard deviation, and proportions) will be used to describe the AYA cancer patient population and incidence of impaired health outcomes for the total population and per subgroup (e.g., tumor type, age group).

##### Exploratory

We will carry out repeated analyses using multilevel linear mixed models, which accounts for the intra-patient dependency of the repeated measures. Missing outcomes will be assumed missing at random (MAR). An advantage of multilevel linear mixed models is that all patients can be included in the analyses, regardless of whether they have been missing some follow-up measurements.

Moderation (address, when, or under what circumstances, or for what types of people an effect exists or does not and in what magnitude) and mediation (how a causal effect operates) will be tested according to the recommendations of Hayes et al. [64] using the principles of ordinary least squares regression analysis and conditional process analysis for the combination of moderation and mediation. 

## 3. Discussion

To our knowledge, this is the first European study comprehensively assessing incidence, risk factors, and underlying mechanisms of impaired (age-specific) medical and psychosocial health outcomes in a hitherto neglected group of patients, who play an important role in society. The National Cancer Institute Progress Review Group on AYA oncology recently concluded that access to AYA samples for studies is a challenge. Therefore, one of the most important contributions to AYA oncology research would be pooling of data resources across institutions to include biologic samples and access to individuals during and after treatment. In the era of precision medicine, this will be imperative for advances in AYA oncology. Hence, we share our protocol to enable collaboration in due course with international initiatives and registries to increase the sample size. The COMPRAYA study and research infrastructure is a very timely solution. It is also fits with statements made in the recent position paper from the AYA working group of ESMO and SIOPE [30]. Results will make it possible to identify patients at risk of poor outcomes and support them by providing timely and age-adjusted information and education about risk factors and offer age-specific (supportive) care.

### 3.1. Limitations

#### 3.1.1. Anticipated Uptake and Representativeness Study Sample

The anticipated uptake of 40% is based on previous studies among AYA reporting response rates of 29–52% [44,65,66,67]. Several methods are described in the AYA literature to overcome low response rates: engaging patients; invitations originating from patients’ own medical institutions; use of in-person contact; reminder calls; use AYA-adjusted communication channels, including advertisement via social media; using plain instead of colorful questionnaires and patient-preferred pencil and paper, rather than online, surveys [68]. We incorporated these strategies in COMPRAYA and will make use of the available nationwide AYA ‘Young and Cancer’ Care Network to enhance recruitment. In addition, patients will be involved as partners in research via an AYA council. By treating the patient as a partner in research it will become easier to identify patients’ biomedical and psychosocial needs and address their needs according to their expectations. We actively involved 11 AYA cancer patients in writing the project grant (what research questions do they want to get answered?). Their needs and wishes are adequately reflected in the final study protocol. The patients represented in the COMPRAYA AYA council supported the research team with decision-making on the outcome measures, composing study materials and creating the recruitment strategy, and will be involved in the development of a COMPRAYA platform (described below). Furthermore, anonymous basic clinical and sociodemographic data from non-respondents will be available from the NCR, which will give an indication of the generalizability of COMPRAYA findings and will help to identify hard-to-reach groups.

#### 3.1.2. Logistical Challenges

Given the heterogeneity of AYAs with regard to cancer diagnoses and related healthcare specialists, the identification of new AYA cancer patients can be a complicated task. The usage of the NCR as a sampling frame for COMPRAYA will facilitate recruitment. The appointment of dedicated COMPRAYA research nurses in the participating hospitals, who will work alongside the healthcare team, will ensure that all eligible patients are invited and guarantees a personal invitation approach. We do acknowledge, however, that the AYA population diagnosed and treated in university medical centers (the current participating centers of COMPRAYA) might not adequately reflect the complete AYA cancer population (patients with more aggressive tumor types and/or rare cancers are more often referred to those (expert) hospitals) and therefore we aim to open COMPRAYA in regional centers when all teething problems in the large centers are solved, to get a fully representative sample with regard to tumor epidemiology.

### 3.2. Strengths and Opportunities

#### 3.2.1. Future Innovative Cohort Multiple-Randomized Controlled Trial

We aim to incorporate a cohort multiple-randomized controlled trial (cmRCT) component to the COMPRAYA study. The cmRCT design uses a large observational cohort of people with a specific disease (in this case age at first cancer diagnosis), with regular measurement of outcomes for the whole cohort. Cohorts provide capacity for multiple randomized controlled trials (RCTs) over time, but also for additional observational studies. For each new RCT (e.g., psychosocial self-management intervention or antagonist treatment for prevention of cardiotoxic effects), routine cohort data help to identify those eligible for the trial. Eligible cohort members are then randomly selected to be offered the intervention, and their outcomes are compared with those not randomly selected (those receiving usual care). This innovative study design will facilitate (inter)national collaboration and optimal use of the data we collect from this rare and heterogeneous study population.

#### 3.2.2. Accessibility Data, (Inter)national Collaboration, Continuity Research

Collected data from the COMPRAYA study will be shared and published according to FAIR data stewardship, by making our data findable, accessible, and interoperable, the data will also be reusable. (Inter)national researchers can request (1) collected data for data analysis or (2) to conduct an ancillary (intervention) study where additional data not currently available will be collected (own funding external researchers). By sharing data, future research into risk factors and underlying mechanisms of (age-specific) health outcomes will be facilitated, ensuring the continuity of and collaboration in AYA research. The ultimate goal would be to collaborate internationally, share data, and collect similar data in other countries to create a huge knowledge hub for AYA cancer research. A flexible core outcome set, a consensus-based agreed minimum set of outcomes that should be measured and reported in all studies of a specific population, will be of utmost importance [69]. Up to now most core outcome sets focus on specific diseases and not on age-related outcomes. The establishment of an international standard to align existing and newly developing research and healthcare initiatives would ease implementation and unlock far greater global collaboration to deliver better care at lower cost [70]. We acknowledge that collection of a standard set requires significant upfront investment in information technology and/or data collection resources. Countries and organizations will certainly vary in their readiness to adopt this and therefore a stepwise implementation is recommended. Now that we are in the era of big data, the extremely fast-evolving health information technology infrastructure will contribute to success in building cohorts. These combined, international clinical- and population-based data are required for the development of prevention, screening, and treatment strategies to improve AYA cancer outcomes. Given the enormous amount of data that will then potentially become available, it is worth to consider implementing artificial-intelligence-modelling techniques to answer new research questions.

#### 3.2.3. COMPRAYA Patient Platform

Finally, next to the COMPRAYA study and research infrastructure, an online platform will be created with and for AYA cancer patients. Patients reported that they want to receive feedback on their outcomes, reflect on a difficult period in life, and share experiences, access to relevant information, and new interventions. The COMPRAYA platform will provide age-specific information (e.g., on return to work), support (e.g., decision-making tools and communication strategies) and potential interventions (e.g., online community). 

## 4. Conclusions

To conclude, the systematic and comprehensive collection and analysis of patient, tumor, and treatment characteristics and physical and psychosocial outcomes of AYA cancer patients over time will support the development of evidence-based AYA care programs and guidelines. Future international collaboration is of utmost importance.

## Figures and Tables

**Table 1:** Schedule of assessments.

	Screening/ Baseline	Follow-Up 1 Year	Follow-Up 2 Years	Follow-Up 3,4,6,7,8,9 Years	Follow-Up 5 Years	Follow-Up 10 Years
		(profiles only)		(profiles only)		
Informed consent	X					
Background characteristics ^1^	X	X	X	X	X	X
Clinical and treatment characteristics	X		X		X	X
Genetic background and family history/special phenotypic features	X					
Lifestyle and other environmental exposures ^2^	X		X		X	X
Medical history/conditions	X		X		X	X
Physical examination/vital parameters ^3^	X		X		X	
Blood sample + standard questionnaire	X		X		X	
Feces sample + standard questionnaire ^4^	X		X		X	
Hair sample + standard questionnaire	X		X		X	
Questionnaires						
Impact cancer ^5^	X	X	X	X	X	X
Health-related quality of life ^6^	X	X	X	X	X	X
Psychological distress ^7^	X	X	X	X	X	X
Psychosocial characteristics ^8^	X		X	X	X	X
Costs related to productivity and medical consumption ^9^	X	X	X	X	X	X
Food-intake diaries	X	X	X		X	X
Survival and registry linkage	---------------------------------------------------------------------------------->					

^1^ Age, gender, ethnicity, postal code, partner status, living situation, education, employment, income, siblings, parenthood; ^2^ smoking, alcohol, drugs, exercise (SQUASH), nutrition, sun behavior, sedentary behavior, CAM; ^3^ blood pressure, heart rate, BMI, grip strength, bio-impedance measurement; ^4^ feces only collected in three hospitals; ^5^ 18-item Life Impact Checklist; ^6^ EORTC QLQ-C30; EQ5D; ^7^ HADS; ^8^ BIPQ, CERQ, BRS, PAQ, SPIRIT; ^9^ iMCQ, IPCQ, PRODISQ.

## Data Availability

Data will be available on request due to restrictions (e.g., privacy or ethical).

## References

[1] Meeneghan M.R., Wood W.A. (2014). Challenges for cancer care delivery to adolescents and young adults: Present and future. Acta Haematol..

[2] Adolescent and Young Adult Oncology Review Group (2006). Closing the Gap: Research and Care Imperatives for Adolescents and Young Adults with Cancer.

[3] Lewis D.R., Seibel N.L., Smith A.W., Stedman M.R. (2014). Adolescent and young adult cancer survival. J. Natl. Cancer Inst. Monogr..

[4] Smith A.W., Seibel N.L., Lewis D.R., Albritton K.H., Blair D.F., Blanke C.D., Bleyer W.A., Freyer D.R., Geiger A.M., Hayes-Lattin B. (2016). Next steps for adolescent and young adult oncology workshop: An update on progress and recommendations for the future. Cancer Am. Cancer Soc..

[5] Marshall S., Grinyer A., Limmer M. (2018). The ‘lost tribe’ reconsidered: Teenagers and young adults treated for cancer in adult settings in the UK. Eur. J. Oncol. Nurs..

[6] Michelagnoli M.P., Pritchard J., Phillips M.B. (2003). Adolescent oncology—A homeland for the “lost tribe”. Eur. J. Cancer.

[7] (2021). Netherlands Cancer Registry. www.cijfersoverkanker.nl.

[8] Aben K.K., van Gaal C., van Gils N.A., van der Graaf W.T., Zielhuis G.A. (2012). Cancer in adolescents and young adults (15–29 years): A population-based study in the Netherlands 1989–2009. Acta Oncol..

[9] Bleyer A., Barr R., Hayes-Lattin B., Thomas D., Ellis C., Anderson B., on behalf of the Biology and Clinical Trials Subgroups of the US National Cancer Institute Progress Review Group in Adolescent and Young Adult Oncology (2008). The distinctive biology of cancer in adolescents and young adults. Nat. Rev. Cancer.

[10] Trama A., Botta L., Foschi R., Ferrari A., Stiller C., Desandes E., Maule M.M., Merletti F., Gatta G., Group E.-W. (2016). Survival of European adolescents and young adults diagnosed with cancer in 2000–2007: Population-based data from EUROCARE-5. Lancet Oncol..

[11] Keegan T.H., Ries L.A., Barr R.D., Geiger A.M., Dahlke D.V., Pollock B.H., Bleyer W.A., for the National Cancer Institute Next Steps for Adolescent and Young Adult Oncology Epidemiology Working Group (2016). Comparison of cancer survival trends in the United States of adolescents and young adults with those in children and older adults. Cancer.

[12] Bleyer A., Budd T., Montello M. (2006). Adolescents and young adults with cancer: The scope of the problem and criticality of clinical trials. Cancer.

[13] Barr R.D., Ries L.A., Lewis D.R., Harlan L.C., Keegan T.H., Pollock B.H., Bleyer W.A., for the US National Cancer Institute Science of Adolescent and Young Adult Oncology Epidemiology Working Group (2016). Incidence and incidence trends of the most frequent cancers in adolescent and young adult Americans, including “nonmalignant/noninvasive” tumors. Cancer.

[14] Trama A., Bernasconi A., McCabe M.G., Guevara M., Gatta G., Botta L., Group R.A.W., Ries L., Bleyer A. (2019). Is the cancer survival improvement in European and American adolescent and young adults still lagging behind that in children?. Pediatr. Blood Cancer.

[15] van der Meer D.J., Karim-Kos H.E., van der Mark M., Aben K.K.H., Bijlsma R.M., Rijneveld A.W., van der Graaf W.T.A., Husson O. (2020). Incidence, Survival, and Mortality Trends of Cancers Diagnosed in Adolescents and Young Adults (15–39 Years): A Population-Based Study in The Netherlands 1990–2016. Cancers.

[16] Burgers V.W.G., van der Graaf W.T.A., van der Meer D.J., McCabe M.G., Rijneveld A.W., van den Bent M.J., Husson O. (2021). Adolescents and Young Adults Living with an Uncertain or Poor Cancer Prognosis: The “New” Lost Tribe. J. Natl. Compr. Cancer Netw..

[17] De R., Sutradhar R., Kurdyak P., Aktar S., Pole J.D., Baxter N., Nathan P.C., Gupta S. (2021). Incidence and Predictors of Mental Health Outcomes Among Survivors of Adolescent and Young Adult Cancer: A Population-Based Study Using the IMPACT Cohort. J. Clin. Oncol..

[18] Fidler M.M., Reulen R.C., Bright C.J., Henson K.E., Kelly J.S., Jenney M., Ng A., Whelan J., Winter D.L., Frobisher C. (2018). Respiratory mortality of childhood, adolescent and young adult cancer survivors. Thorax.

[19] Keegan T.H.M., Bleyer A., Rosenberg A.S., Li Q., Goldfarb M. (2017). Second Primary Malignant Neoplasms and Survival in Adolescent and Young Adult Cancer Survivors. JAMA Oncol..

[20] Keegan T.H.M., Li Q., Steele A., Alvarez E.M., Brunson A., Flowers C.R., Glaser S.L., Wun T. (2018). Sociodemographic disparities in the occurrence of medical conditions among adolescent and young adult Hodgkin lymphoma survivors. Cancer Causes Control.

[21] John T.D., Sender L.S., Bota D.A. (2016). Cognitive Impairment in Survivors of Adolescent and Early Young Adult Onset Non-CNS Cancers: Does Chemotherapy Play a Role?. J. Adolesc. Young Adult Oncol..

[22] Hayashi R.J. (2019). Adolescent and young adult cancer survivorship: The new frontier for investigation. Cancer.

[23] Woodward E., Jessop M., Glaser A., Stark D. (2011). Late effects in survivors of teenage and young adult cancer: Does age matter?. Ann. Oncol..

[24] Dommett R.M., Redaniel M.T., Stevens M.C., Hamilton W., Martin R.M. (2013). Features of cancer in teenagers and young adults in primary care: A population-based nested case-control study. Br. J. Cancer.

[25] Fern L.A., Birch R., Whelan J., Cooke M., Sutton S., Neal R.D., Gerrand C., Hubbard G., Smith S., Lethaby C. (2013). Why can’t we improve the timeliness of cancer diagnosis in children, teenagers, and young adults?. BMJ.

[26] Tricoli J.V., Blair D.G., Anders C.K., Bleyer W.A., Boardman L.A., Khan J., Kummar S., Hayes-Lattin B., Hunger S.P., Merchant M. (2016). Biologic and clinical characteristics of adolescent and young adult cancers: Acute lymphoblastic leukemia, colorectal cancer, breast cancer, melanoma, and sarcoma. Cancer.

[27] Tricoli J.V., Seibel N.L., Blair D.G., Albritton K., Hayes-Lattin B. (2011). Unique characteristics of adolescent and young adult acute lymphoblastic leukemia, breast cancer, and colon cancer. J. Natl. Cancer Inst..

[28] Potosky A.L., Harlan L.C., Albritton K., Cress R.D., Friedman D.L., Hamilton A.S., Kato I., Keegan T.H., Keel G., Schwartz S.M. (2014). Use of appropriate initial treatment among adolescents and young adults with cancer. J. Natl. Cancer Inst..

[29] Barr R.D., Ferrari A., Ries L., Whelan J., Bleyer W.A. (2016). Cancer in Adolescents and Young Adults: A Narrative Review of the Current Status and a View of the Future. JAMA Pediatr..

[30] Ferrari A., Stark D., Peccatori F.A., Fern L., Laurence V., Gaspar N., Bozovic-Spasojevic I., Smith O., de Munter J., Derwich K. (2021). Adolescents and young adults (AYA) with cancer: A position paper from the AYA Working Group of the European Society for Medical Oncology (ESMO) and the European Society for Paediatric Oncology (SIOPE). ESMO Open.

[31] Zebrack B.J. (2011). Psychological, social, and behavioral issues for young adults with cancer. Cancer-Am. Cancer Soc..

[32] Zebrack B., Isaacson S. (2012). Psychosocial care of adolescent and young adult patients with cancer and survivors. J. Clin. Oncol..

[33] Sodergren S.C., Husson O., Rohde G.E., Tomasewska I.M., Vivat B., Yarom N., Griffiths H., Darlington A.S. (2018). A Life Put on Pause: An Exploration of the Health-Related Quality of Life Issues Relevant to Adolescents and Young Adults with Cancer. J. Adolesc. Young Adult Oncol..

[34] Husson O., Huijgens P.C., van der Graaf W.T.A. (2018). Psychosocial challenges and health-related quality of life of adolescents and young adults with hematologic malignancies. Blood.

[35] Husson O., Zebrack B.J., Block R., Embry L., Aguilar C., Hayes-Lattin B., Cole S. (2017). Health-Related Quality of Life in Adolescent and Young Adult Patients with Cancer: A Longitudinal Study. J. Clin. Oncol..

[36] Greup S.R., Kaal S.E.J., Jansen R., Manten-Horst E., Thong M.S.Y., van der Graaf W.T.A., Prins J.B., Husson O. (2018). Post-Traumatic Growth and Resilience in Adolescent and Young Adult Cancer Patients: An Overview. J. Adolesc. Young Adult Oncol..

[37] Harju E., Roser K., Dehler S., Michel G. (2018). Health-related quality of life in adolescent and young adult cancer survivors. Support. Care Cancer.

[38] Robison L.L., Mertens A.C., Boice J.D., Breslow N.E., Donaldson S.S., Green D.M., Li F.P., Meadows A.T., Mulvihill J.J., Neglia J.P. (2002). Study design and cohort characteristics of the Childhood Cancer Survivor Study: A multi-institutional collaborative project. Med. Pediatr. Oncol..

[39] Teepen J.C., van Leeuwen F.E., Tissing W.J., van Dulmen-den Broeder E., van den Heuvel-Eibrink M.M., van der Pal H.J., Loonen J.J., Bresters D., Versluys B., Neggers S. (2017). Long-Term Risk of Subsequent Malignant Neoplasms After Treatment of Childhood Cancer in the DCOG LATER Study Cohort: Role of Chemotherapy. J. Clin. Oncol..

[40] van de Poll-Franse L.V., Horevoorts N., van Eenbergen M., Denollet J., Roukema J.A., Aaronson N.K., Vingerhoets A., Coebergh J.W., de Vries J., Essink-Bot M.L. (2011). The Patient Reported Outcomes Following Initial treatment and Long term Evaluation of Survivorship registry: Scope, rationale and design of an infrastructure for the study of physical and psychosocial outcomes in cancer survivorship cohorts. Eur. J. Cancer.

[41] Wilson I.B., Cleary P.D. (1995). Linking clinical variables with health-related quality of life. A conceptual model of patient outcomes. JAMA.

[42] Ferrans C.E., Zerwic J.J., Wilbur J.E., Larson J.L. (2005). Conceptual model of health-related quality of life. J. Nurs. Sch..

[43] Sprangers M.A., Sloan J.A., Barsevick A., Chauhan C., Dueck A.C., Raat H., Shi Q., Van Noorden C.J., Consortium G. (2010). Scientific imperatives, clinical implications, and theoretical underpinnings for the investigation of the relationship between genetic variables and patient-reported quality-of-life outcomes. Qual. Life Res..

[44] Bellizzi K.M., Smith A., Schmidt S., Keegan T.H., Zebrack B., Lynch C.F., Deapen D., Shnorhavorian M., Tompkins B.J., Simon M. (2012). Positive and negative psychosocial impact of being diagnosed with cancer as an adolescent or young adult. Cancer.

[45] Ganz P.A., Desmond K.A., Leedham B., Rowland J.H., Meyerowitz B.E., Belin T.R. (2002). Quality of life in long-term, disease-free survivors of breast cancer: A follow-up study. J. Natl. Cancer Inst..

[46] Zebrack B.J., Mills J., Weitzman T.S. (2007). Health and supportive care needs of young adult cancer patients and survivors. J. Cancer Surviv. Res. Pract..

[47] Zebrack B. (2009). Developing a new instrument to assess the impact of cancer in young adult survivors of childhood cancer. J. Cancer Surviv. Res. Pract..

[48] Aaronson N.K., Ahmedzai S., Bergman B., Bullinger M., Cull A., Duez N.J., Filiberti A., Flechtner H., Fleishman S.B., de Haes J.C. (1993). The European Organization for Research and Treatment of Cancer QLQ-C30: A quality-of-life instrument for use in international clinical trials in oncology. J. Natl. Cancer Inst..

[49] Herdman M., Gudex C., Lloyd A., Janssen M., Kind P., Parkin D., Bonsel G., Badia X. (2011). Development and preliminary testing of the new five-level version of EQ-5D (EQ-5D-5L). Qual. Life Res..

[50] Zigmond A.S., Snaith R.P. (1983). The hospital anxiety and depression scale. Acta Psychiatr. Scand..

[51] Bouwmans C., Krol M., Brouwer W., Severens J.L., Koopmanschap M.A., Hakkaart L. (2014). IMTA Productivity Cost Questionnaire (IPCQ). Value Health.

[52] Koopmanschap M.A. (2005). PRODISQ: A modular questionnaire on productivity and disease for economic evaluation studies. Expert Rev. Pharm. Outcomes Res..

[53] van Herk-Sukel M.P., van de Poll-Franse L.V., Lemmens V.E., Vreugdenhil G., Pruijt J.F., Coebergh J.W., Herings R.M. (2010). New opportunities for drug outcomes research in cancer patients: The linkage of the Eindhoven Cancer Registry and the PHARMO Record Linkage System. Eur. J. Cancer.

[54] Sperling C.D., Petersen G.S., Holge-Hazelton B., Graugaard C., Winther J.F., Gudmundsdottir T., Ahrensberg J., Schmiegelow K., Boisen K.A., Olsen P.R. (2016). Being Young and Getting Cancer: Development of a Questionnaire Reflecting the Needs and Experiences of Adolescents and Young Adults with Cancer. J. Adolesc. Young Adult Oncol..

[55] Taylor R.M., Fern L.A., Solanki A., Hooker L., Carluccio A., Pye J., Jeans D., Frere-Smith T., Gibson F., Barber J. (2015). Development and validation of the BRIGHTLIGHT Survey, a patient-reported experience measure for young people with cancer. Health Qual. Life Outcomes.

[56] Garnefski N., Kraaij V. (2006). Cognitive emotion regulation questionnaire–development of a short 18-item version (CERQ-short). Personal. Individ. Differ..

[57] Smith B., Dalen J., Wiggins K., Tooley E., Christopher P., Bernard J. (2008). The Brief Resilience Scale: Assessing the Ability to Bounce Back. Int. J. Behav. Med..

[58] Broadbent E., Petrie K.J., Main J., Weinman J. (2006). The brief illness perception questionnaire. J. Psychosom. Res..

[59] Vernooij-Dassen M.J., Osse B.H., Schade E., Grol R.P. (2005). Patient autonomy problems in palliative care: Systematic development and evaluation of a questionnaire. J. Pain Symptom Manag..

[60] Smeets W. (2010). Het spirituele aspect in het detecteren van psychosociale behoeften in de oncologische praktijk. Psyche Geloof.

[61] Wendel-Vos G.C., Schuit A.J., Saris W.H., Kromhout D. (2003). Reproducibility and relative validity of the short questionnaire to assess health-enhancing physical activity. J. Clin. Epidemiol..

[62] Wright K.P., Drake A.L., Frey D.J., Fleshner M., Desouza C.A., Gronfier C., Czeisler C.A. (2015). Influence of sleep deprivation and circadian misalignment on cortisol, inflammatory markers, and cytokine balance. Brain Behav. Immun..

[63] Field A. (2010). Discovering Statistics Using SAS.

[64] Hayes A.F., Rockwood N.J. (2017). Regression-based statistical mediation and moderation analysis in clinical research: Observations, recommendations, and implementation. Behav. Res. Ther..

[65] Husson O., Zebrack B.J. (2017). Perceived impact of cancer among adolescents and young adults: Relationship with health-related quality of life and distress. Psychooncology.

[66] Murnane A., Gough K., Thompson K., Holland L., Conyers R. (2015). Adolescents and young adult cancer survivors: Exercise habits, quality of life and physical activity preferences. Support. Care Cancer.

[67] Kaal S.E.J., Husson O., van Duivenboden S., Jansen R., Manten-Horst E., Servaes P., Prins J.B., van den Berg S.W., van der Graaf W.T.A. (2017). Empowerment in adolescents and young adults with cancer: Relationship with health-related quality of life. Cancer.

[68] Rosenberg A.R., Bona K., Wharton C.M., Bradford M., Shaffer M.L., Wolfe J., Baker K.S. (2016). Adolescent and Young Adult Patient Engagement and Participation in Survey-Based Research: A Report From the “Resilience in Adolescents and Young Adults with Cancer” Study. Pediatr. Blood Cancer.

[69] Husson O., Sodergren S.C., Darlington A.S. (2021). The importance of a collaborative health-related quality of life measurement strategy for adolescents and young adults with cancer. Cancer.

[70] Dodd S., Harman N., Taske N., Minchin M., Tan T., Williamson P.R. (2020). Core outcome sets through the healthcare ecosystem: The case of type 2 diabetes mellitus. Trials.

